# Effect of Heat Treatment on the Quality and Soft Rot Resistance of Sweet Potato during Long-Term Storage

**DOI:** 10.3390/foods12234352

**Published:** 2023-12-02

**Authors:** Jifeng Wu, Jingzhen Zhang, Wenrong Ni, Ximing Xu, Melvin Sidikie George, Guoquan Lu

**Affiliations:** 1 Institute of Root and Tuber Crops, The Key Laboratory for Quality Improvement of Agricultural Products of Zhejiang Province, College of Advanced Agricultural Sciences, Zhejiang A&F University, Hangzhou 311300, China; jifeng@stu.zafu.edu.cn (J.W.); jingzhen0118@stu.zafu.edu.cn (J.Z.); niwenrong_ntc@163.com (W.N.); xuximing@zafu.edu.cn (X.X.); 2 Crop Science Department, Njala University, Njala Campus, Private Mail Bag, Freetown 999127, Sierra Leone; mgeorge@njala.edu.sl

**Keywords:** *Ipomoea batatas*, heat treatment, soft rot, quality characteristic

## Abstract

Heat treatment is a widely applied technique in the preservation of fruits and vegetables, effectively addressing issues such as disease management, rot prevention, and browning. In this study, we investigated the impact of heat treatment at 35 °C for 24 h on the quality characteristics and disease resistance of two sweet potato varieties, P32/P (*Ipomoea batatas* (L.) Lam. cv ‘Pushu13’) and Xinxiang (*Ipomoea batatas* (L.) Lam. cv ‘Xinxiang’). The growth in vitro and reproduction of *Rhizopus stolonifer* were significantly inhibited at 35 °C. However, it resumed when returned to suitable growth conditions. The heat treatment (at 35 °C for 24 h) was found to mitigate nutrient loss during storage while enhancing the structural characteristics and free radical scavenging capacity of sweet potato. Additionally, it led to increased enzyme activities for APX, PPO, and POD, alongside decreased activities for Cx and PG, thereby enhancing the disease resistance of sweet potato against soft rot. As a result, the heat treatment provided a theoretical basis for the prevention of sweet potato soft rot and had guiding significance for improving the resistance against sweet potato soft rot.

## 1. Introduction 

Sweet potato soft rot, caused by parasitic fungi (*Rhizopus stolonifer*), has emerged as a prominent contributor to postharvest losses in sweet potato (*Ipomoea batatas* (L.) Lam) [1]. Presently, synthetic fungicides constitute the primary means to mitigate Rhizopus soft rot before storage [2]. Although chemical fungicides have demonstrated efficacy in controlling various postharvest diseases in fruits and vegetables, their application presents a litany of concerns, including the development of pathogen resistance, environmental hazards, chemical residue, and potential adverse effects on human health [3]. 

Additionally, some nutrients in sweet potato could be lost during storage. The nutritional quality of the tuberous root of sweet potato refers to its various nutrients, such as starch, soluble sugar, carotene, anthocyanin, flavone, protein, mineral elements, and vitamins [4]. Starch is the main nutrient of sweet potato; it can convert into soluble sugar during storage, and its content (50–80% of dry weight) reduces with time [5]. The soluble sugar content (1.2–24.4% of dry weight) increased first and then decreased during storage because a large amount of soluble sugar was consumed by the respiration of sweet potato in later stages [6]. Sweet potato is rich in protein (1.73–9.14% of dry weight), containing 18 kinds of amino acids [7], which followed a trend of first increasing, next decreasing, and then increasing during a 160 day storage period [8]. Flavone has antioxidant, anti-inflammatory, and anticancer effects [9]. It is a kind of trace element that shows a fluctuating upward trend during storage [10]. Consequently, there is a pressing need to identify more environmentally friendly approaches to control sweet potato soft rot and maintain nutritional quality.

Postharvest heat treatment stands out as a green, safe, and efficient physical preservation method [11] that encompasses a wide spectrum of treatments characterized by varying temperature ranges (35~60 °C), exposure durations (ranging from seconds to days), and heating methodologies (including microwave, infrared, hot vapor, hot air, and hot water) [12,13,14,15]. Heat treatment has been used in the postpartum storage and preservation of fruits and vegetables, to control insect pests [16], as decay control [17], and to reduce chilling injury [18], browning [19], and senescence [20].

Furthermore, proper heat treatment offers advantages that encompass non-toxicity, an absence of residues, preservation of nutritional quality, and flavor enhancement, rendering it a promising method for the storage of fruits and vegetables [21] which has been demonstrated across a range of produce, including banana [22], peach [23,24,25], melon [26,27], apple [28,29,30], pepper [31], tomato [32,33,34], cabbage [35,36], and sweet potato [37,38]. For instance, treatment at 46 °C for 30 min before low-temperature storage effectively reduced chilling injury during refrigeration and preserved the quality of honey peaches [25]. Morgado et al. [39] discovered that soaking melon fruits in hot water (at 50 °C for 30 min) temporarily reduces the respiration rate of fresh-cut melons, delays decay and deterioration, and increases beta-carotene content. Heat treatment at 45 °C for 3 h maintains the total phenolic compounds and antioxidant capacity of ‘Red Fuji’ apples [28]. Mild heat treatment (at 45 °C for 3 min) of freshly cut peppers significantly reduces soft rot incidence, delays pectin dissolution and softening, and prevents membrane leakage [40]. Heat shock to tomatoes for 24 or 48 h maintains their quality properties and extends their shelf life during low temperature storage [33]. Moreover, heat treatment with a soaking solvent effectively enhances the antioxidant properties of sweet potato cultivars [41]. Thus, these studies substantiate that proper heat treatment can effectively preserve quality and mitigate the decay rate of fruits and vegetables.

Therefore, it is of great practical significance to study heat treatment for the prevention of soft rot in sweet potato during long-term storage and the preservation of nutritional quality. In this study, we selected two sweet potato varieties with different resistance to soft rot and an appropriate pre-storage heat treatment condition (at 35 °C for 24 h) based on previous experiments concerning sweet potato soft rot prevention. We also investigated the impact of heat treatment on the growth and reproduction of *R. stolonifer* in vitro, the nutritional and textural characteristics of healthy sweet potato, and the physiological mechanisms underlying sweet potato infection by *R. stolonifer*. It is hoped that the physical method of heat treatment can provide a theoretical basis for the follow-up study of the prevention and control of sweet potato soft rot during postharvest storage.

## 2. Materials and Methods 

### 2.1. Experimental Materials and Treatments 

Two fresh sweet potato cultivars, P32/P (*Ipomoea batatas* (L.) Lam. cv ‘Pushu13’) and Xinxiang (*Ipomoea batatas* (L.) Lam. cv ‘Xinxiang’), bred by Puning Agricultural Science and the Crop Research Institute of Zhejiang Academy of Agricultural Sciences, respectively, were harvested by hand 120 days after planting in the agricultural field area of Zhejiang A & F University in June 2021 and selected to investigate antifungal effects in this experiment. *R. stolonifer* was provided by Xuzhou Institute of Agricultural Sciences, Jiangsu Province, China.

Sweet potato tuberous roots that were uniform in size and appearance without physical injury or infection were chosen and randomly divided into two groups. They were first stored at 28 °C and 85% relative humidity (RH) for 24 h (CK) and at 35 °C and 85% RH for 24 h (HT), then cooled at 25 °C and 85% RH for 24 h before finally being stored at 13 °C and 85% RH for 60 days.

### 2.2. Sample Preparation 

Fresh root sample preparation: Using the four-part sampling method, firstly, take two diagonal pieces of each root block. Secondly, put them into the dicing machine to fully chop and mix them. Finally, freeze them in liquid nitrogen; then, keep them in the refrigerator at −80 °C for further use.

Root powder sample preparation: Includes cutting with a slicer, liquid nitrogen freezing, transfer to a freeze-dryer for −80 °C freeze-drying, grinding into powder with a hammer cyclone mill (JXFM110, Daji Electric Instrument Co., Ltd., Hangzhou, China), sifting through 80 mesh sieve, and low-temperature dry storage, avoiding light. Each treatment was prepared by randomly selecting three roots of the same size, which were washed with tap water and dried, and three biological replicates.

### 2.3. Antifungal Activity of Heat Treatment on R. stolonifer In Vitro 

For the mycelial growth experiment, the inhibitory effects of the HT on *R. stolonifer* were tested according to the method of Arrebola et al. [42]. First, 15 mL of PDA was used to fill Petri plates (90 mm diameter). Hyphal mass discs of *R. stolonifer* were put in the center of each PDA plate for culturing, while three treatments were applied in artificial climate boxes (SAFE, Saifu Experimental Instrument Co., Ltd., Ningbo, China) under 85% RH as follows:

(1) Control A (CK): PDA plates were incubated at 28 °C for 5 days.
(2) Treatment B: PDA plates were incubated at 35 °C for 1 day, followed by incubation at 28 °C for 4 more days.
(3) Treatment C: PDA plates were first incubated at 28 °C for 1 day, then at 35 °C for 1 day, and finally at 28 °C for 3 more days. The colony diameter was measured and averaged after 1, 2, 3, 4 and 5 days by considering two perpendicular diameters for each plate, thus assessing the inhibitory effect of each treatment on mycelium growth.

### 2.4. Resistance to Soft Rot of Sweet Potato 

The resistance of sweet potato tuberous roots to soft rot was determined as described by Yang et al. [43]. The sweet potato tuberous roots (taking three from each treatment) were selected from different storage periods (at 0, 7, 15, 30, 45 and 60 days) and stored at 13 °C and 85% RH. Three sweet potato chips with a thickness of about 8 mm were cut from the middle of each root and inoculated with *R. stolonifer* in their centers, then placed in a sterilized Petri dish with wet filter paper. Finally, the chips with *R. stolonifer* were cultured in an artificial climate box at 26 °C and 85% RH for 21 h. The degree of soft rot was assessed by judging the diameter of disease spots, in order to investigate the phenotypic effect of heat treatment on the resistance of sweet potato to soft rot.

### 2.5. Nutritional Quality Indexes 

#### 2.5.1. Flavone Determination

The content of flavone was determined by referring to Mohammed et al. [44]. Simply, a mixture of root powder sample extract (1.0 mL), 5% NaNO_2_ solution (300 μL) and 10% aluminum chloride (300 μL) was incubated at 25 °C for 5 min. Then, 1.0 mol/L sodium hydroxide (2 mL) was added to the mixture. The mixture was adjusted to a final volume of 10 mL with pure water and mixed thoroughly using a vortex. The absorbance was measured at 510 nm (A_510_) by a spectrophotometer (T6-New Century, General instrument Co., Ltd., Beijing, China).

#### 2.5.2. Starch and Protein Determination

The determination of starch content was based on Takac et al. [45], while the protein content was determined as described by Dong et al. [46]. Both the content of starch and protein in root powder sample of sweet potato were evaluated by near infrared spectroscopy (NIRS) with a FOSS Analytical A/S (DS2500, FOSS Company, Hillerød, Denmark). Each measurement was repeated three times. 

#### 2.5.3. Soluble Sugar Determination

The content of soluble sugar was determined according to Aung et al. [47], using the anthrone-H_2_S0_4_ method. Briefly, root powder sample (0.1 g) was put into a 15 mL tube with 80% ethanol (10 mL). The mixture was incubated at 80 °C for 30 min, then at room temperature for 5 min before centrifugation at 4000 rpm for 10 min. The supernatant was decanted and mixed with 80% ethanol (10 mL) for a second extraction before dilution to a final volume of 25 mL with 80% ethanol. Subsequently, the extract solution (100 μL) was mixed with an equal amount of pure water and anthrone solution (3.0 mL) before incubation at 100 °C for 10 min. The absorbance was measured with a spectrophotometer at 620 nm (A_620_).

### 2.6. Sweet Potato Tuberous Root Texture 

Texture profile analysis (TPA) was used to determine the texture of sweet potato tuberous root referring to the method of Alessandrini et al. [48]. Three samples were randomly selected from each treatment, washed with tap water and dried, and then cut into sweet potato chips with a thickness of 1 cm. The TPA test was conducted by a physical properties analyzer (TMS-PRO, Food Technology Corporation, Dranesville, VA, USA) with the following parameters: Force sensing element range of 500 N, cylindrical probe diameter of 5.0 mm, initial starting force of 0.2 N, speed of 30 min·s^−1^ before puncture, 60 min·s^−1^ during puncture and 60 min·s^−1^ after puncture. The compression ratio was set at 60%, with a retention time of 0 s, and each treatment was repeated 12 times. The calculation of TPA test parameters was performed according to Figure S1 in the Supplementary Materials section.

### 2.7. Antioxidant Enzyme Activity 

Enzyme extract: Fresh root sample (1 g) was added with 1.0 mL phosphate buffer (0.05 M, pH 7.8), which was homogenized by grinding, and then transferred into a centrifuge tube (15 mL), with 4 mL phosphate buffer finally added. The mixture was centrifuged at 10,000 rpm (4 °C) for 10 min, and the supernatant was stored in the refrigerator at 4 °C for analysis.

The superoxide dismutase (SOD) activity was determined by the method of Nadiia et al. [49]. The reaction mixture consisted of enzyme extract (100 μL), 2.7 mL of phosphate buffered saline (PBS) and pure water (500 μL). As controls, two additional tubes were prepared by adding 2.8 mL of PBS and pure water (500 μL). One tube for each specimen was left in the dark, while another was exposed to the light from white lamps (4000 LX) for 20 min in an artificial climate box at 26 °C. The absorbance was measured at 560 nm (A_560_).

The activity of ascorbate peroxidase (APX) was determined by referring to Apiamu et al. [50]. The reaction mixture included enzyme extract (100 μL), 7.5 mM ascorbate (100 μL), 0.05 M phosphate buffer (2700 μL) and hydrogen peroxide (100 μL). Immediately after mixing, the absorbance at 290 nm (A_290_) was recorded every 10 s for 1 min. 

The activity of peroxidase (POD) was determined by the method of Yang et al. [51]. A mixture of 1.5% guaiacol solution (100 μL), enzyme extract (100 μL), 300 uM H_2_O_2_ solution (100 μL) and PBS (2700 μL) was prepared, and immediately after, the absorbance at 470 nm (A_470_) was recorded every 10 s for 1 min. The activity of polyphenol oxidase (PPO) was determined according to Torres et al. [52]. Enzyme extract (1.0 mL) was mixed with 3.9 mL of 100 mM sodium phosphate buffer (pH 6.5) and 100 mM catechol (1.0 mL). Immediately after mixing, the absorbance at 420 nm (A_420_) was recorded every 10 s for 1 min. The above methods were modified.

The determination of the 2,2-diphenyl-1-picrylhydrazyl (DPPH) radical scavenging rate was based on Zhang et al. [53] with slight modification. Briefly, 0.1 mL fresh root sample extract or deionized water (control) was added to 1.0 mL 0.1 M DPPH and 0.9 mL 50 mM Tris-HCl buffer (pH 7.4), mixed thoroughly, and incubated at room temperature for 30 min. The absorbance was measured at 517 nm (A_517_). The DPPH radical scavenging activity was calculated according to the following formula:

DPPH radical scavenging rate (%) = (Absorbance control − Absorbance sample)/Absorbance control × 100

### 2.8. Cell Wall Degrading Enzyme Activity 

Enzyme extract: Fresh root sample (1 g) was homogenized with pre-cooled 95% ethanol (5 mL) and transferred to a centrifuge tube (15 mL). The mixture was placed at 4 °C for 10 min, then centrifuged at 8000 rpm for 5 min, and the sediment was reserved. This process was repeated twice. Then, 5 mL of pre-cooled extraction buffer (including 1.8 mol/L NaCl) was added to the precipitation and placed at 4 °C for 16 min. After centrifugation, the supernatant was collected and stored at 4 °C for analysis.

The activity of polygalactosidase (PG) was determined as described by Zdunek et al. [54]. A mixture of 50 mmol·L^−1^ pH 5.5 acetate-sodium acetate buffer (1 mL), 10 g·L^−1^ polygalacturonic acid solution (0.5 mL) and enzyme extract (0.5 mL) was incubated at 37 °C for 1 h. Next, 1.5 mL of 3,5-dinitrosalicylic acid (DNS) was immediately added, and the mixture was boiled in a water bath for 5 min. Then, the mixture was cooled to room temperature and diluted to 25 mL with pure water. The absorbance was measured at 540 nm (A_540_).

The activity of β-galactosidase (β-Gal) was determined by the method of González-Andrada et al. [55]. In a test tube (15 mL), 1.5 mL of carboxylmethyl cellulose (CMC) solution and enzyme extract (6.5 mL) were combined and incubated at 37 °C for 1 h. The following steps were the same as those in PG activity determination.

The determination of cellulase (Cx) activity was based on the method outlined by Thomas et al. [56]. A mixture of enzyme extract (0.1 mL), pH 4.5 citric acid buffer (0.5 mL) and 0.4 mL of 2 mM o-Nitrophenyl-β-D-galactopyranoside (ONPG) solution was incubated at 37 °C for 30 min. Then, 0.2 M of pre-cooled sodium carbonate solution (4 mL) was added to terminate the reaction. After cooling, pure water was added to reach a final volume of 25 mL, and the mixture was mixed thoroughly. The absorbance was measured at 405 nm (A_405_). The above methods have been modified.

### 2.9. Statistical Analysis 

Means, standard errors and significance analysis of differences were calculated by SPSS 27.0 software (International Business Machines Corporation, Armonk, NY, USA). Statistical analysis employed one-factor analysis of variance, and Duncan’s method was utilized for mean comparisons within varieties at a significance level of *p* < 0.05. Physical and chemical indexes of the samples were determined with a minimum of three repetitions. Figures in this paper were created by Microsoft Excel 2021(Microsoft Corporation, Redmond, WA, USA) and Adobe Illustrator 2020 software (Adobe Systems Incorporated, San Jose, CA, USA).

## 3. Results and Discussion 

### 3.1. Effects of Heat Treatment on the Growth of R. stolonifer in PDA 

**Figure 1:**
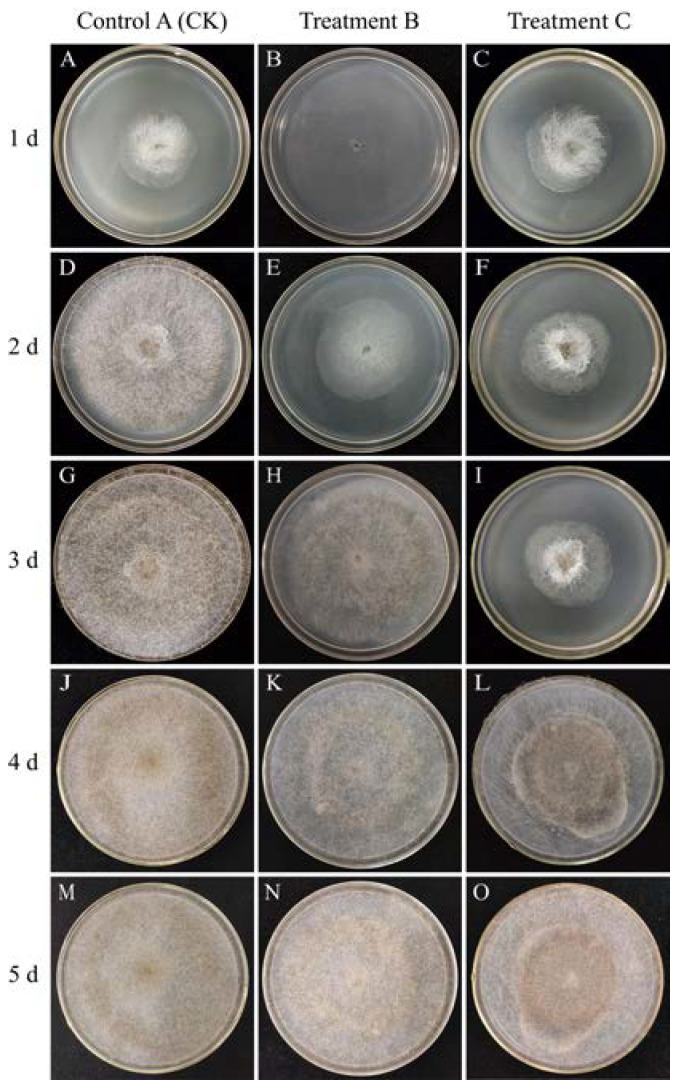
Control A (CK) at 28 °C for 120 h (**A**,**D**,**G**,**J**,**M**), Treatment B at 35 °C for 24 h + 28 °C for 96 h (**B**,**E**,**H**,**K**,**N**), and Treatment C at 28 °C for 24 h + 35 °C for 24 h + 28 °C for 72 h (**C**,**F**,**I**,**L**,**O**) under 85% RH. The morphology and size of the *R. stolonifer* were observed in the PDA medium at 1, 2, 3, 4 and 5 days under different treatments.

The *R. stolonifer* in Treatment B did not grow mycelia and spores on day 1. However, at 28 °C, mycelium began to grow, black spores appeared on day 3, and colonies covered the entire medium by day 4. On the contrary, the *R. stolonifer* in Control A (CK) covered the entire medium with a lot of mycelium and a small number of black spores by day 3. The growth of *R. stolonifer* in Treatment C was similar to that of Control A (CK) on day 1, but from day 2 to day 3, the mycelium started to shrink and gather in the center. Surprisingly, we observed that *R. stolonifer* began to regrow on day 4, and the entire medium was filled with bright white mycelium and black spores by day 5 (Figure 1).

Evidently, heat treatment showed significantly higher antifungal activity against *R. stolonifer* compared to the CK, effectively inhibiting its growth and reproduction. Therefore, we conclude that the heat treatment has the potential to prevent sweet potato soft rot.

### 3.2. Effects of Heat Treatment on Quality Characteristics of Sweet Potato

#### 3.2.1. Nutritional Quality 

**Figure 2:**
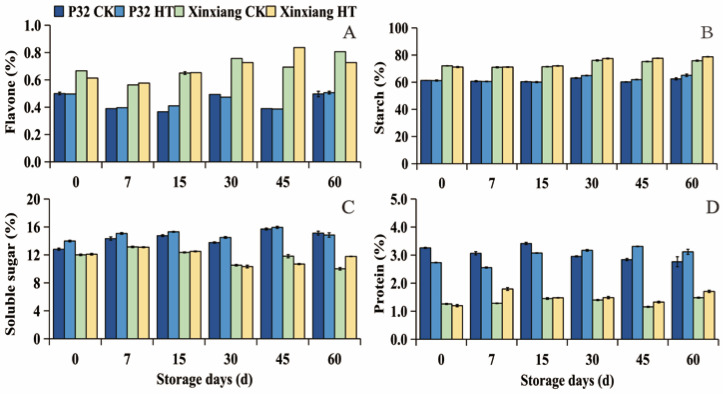
Flavone (**A**), starch (**B**), soluble sugar (**C**) and protein (**D**), in CK0 and HT0 (at 35 °C and 85% RH for 24 h) of P32 and Xinxiang tuber roots stored for 0, 7, 15, 30, 45 and 60 days at 13 °C and 85% RH. CK0 and HT0 mean the sweet potato chips not inoculated with *R. stolonifer*. Significant differences (*p* < 0.05) between means are not indicated by letters, but are presented in the Supplementary Materials section with a three-line table.

The changes in flavone, starch, soluble sugar, and protein contents of sweet potato are shown in Figure 2 and Table S1. These contents were insignificantly affected by heat treatment under storage for 60 days. Flavone is a powerful antioxidant [57]. As shown in Figure 2A, the flavone content of Xinxiang fluctuated between 0.56 (Xinxiang CK0, 7 days)–0.81% (Xinxiang CK0, 60 days). The flavone content of P32 fluctuated between 0.37 (P32 CK0, 15 days)—0.51% (P32 HT0, 60 days). Starch is the primary nutrient in sweet potato tuberous roots. In P32, the starch content increased from 61.30% to 62.44% in CK0 samples, and from 61.20% to 65.09% in HT0 samples. In Xinxiang, the starch content ranged from 72.07% to 75.86% in CK0 samples, and from 71.17% to 78.75% in HT0 samples during the 60-day storage period (Figure 2B). The changes in the soluble sugar content are illustrated in Figure 2C, where P32 exhibited a peak at 45 days (15.7% and 15.94%, respectively). Notably, on day 0, the soluble sugar content in the HT0 group for P32 was 9.00% higher than that in CK0. Both the CK0 and HT0 groups reached their highest values on day 7 (13.14% and 13.10%, respectively). Regarding protein content (Figure 2D), heat treatment led to an increase in Xinxiang over 60 days, peaking on day 7 (1.79%) at nearly 1.40 times higher than the content in CK0 (1.28%). However, for P32, heat treatment showed lower protein content from 0 to 15 days, but higher protein content than in CK0 from 30 to 60 days.

The results indicated that P32 and Xinxiang retain these nutrients more effectively after heat treatment, preventing loss. Previous investigations have found that subjecting peaches to heat at 46 °C for 30 min before refrigeration increased the contents of soluble protein and heat-stable protein (HSP) [25]. The investigations on postharvest heat shock (at 65 °C for 15 min) in sweet potato callus by Lyu [58] found that the heat treatment enhanced the content of starch and soluble sugar in sweet potato during storage. Furthermore, Zhang et al. [59] discovered that Tartary buckwheat sprouts’ flavone content was enhanced by heat shock (at 45 °C for 10 min). In this study, sweet potato protein remained at a stable level during storage, but heat shock significantly increased the protein content at the later stage of storage, which may be related to the expression of heat shock proteins (Hsps) induced by heat treatment [60].

#### 3.2.2. Antioxidant Enzyme Activity 

**Figure 3:**
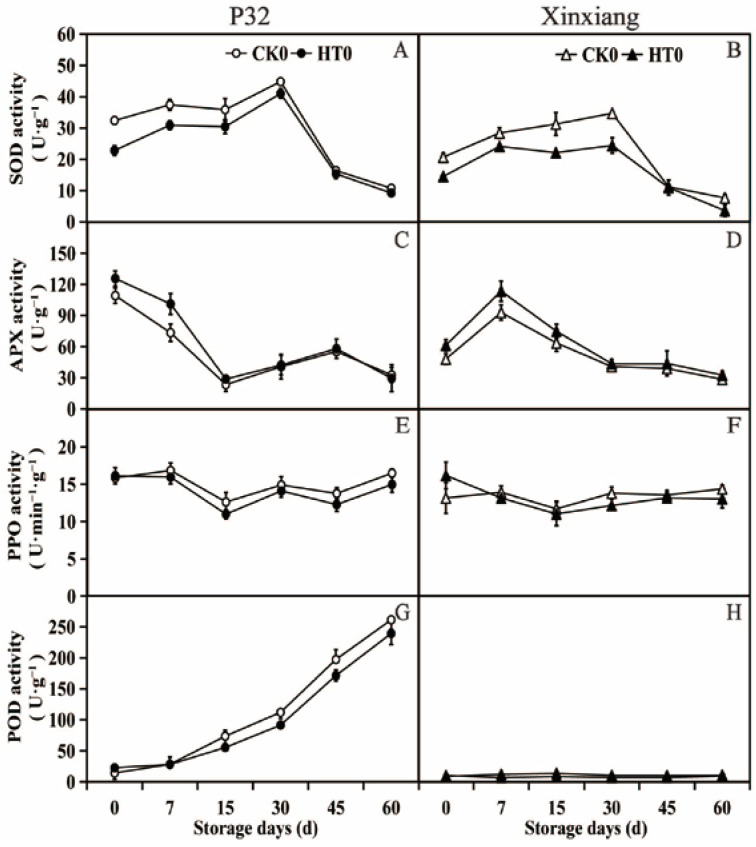
SOD activity (**A**,**B**), APX activity (**C**,**D**), PPO activity (**E**,**F**) and POD activity (**G**,**H**) in P32 and Xinxiang tuberous roots stored for 0, 7, 15, 30, 45 and 60 days at 13 °C and 85% RH. CK0 and HT0 mean the sweet potato chips not inoculated with *R. stolonifer*. Significant differences (*p* < 0.05) between means are not indicated by letters above the broken lines, but are presented in the Supplementary Materials with a three-line table.

To explore whether the development of resistance following heat treatment involved the activation of antioxidant enzymes, the activities of SOD, APX, PPO and POD enzymes were analyzed at different storage times after the heat treatment (Figure 3 and Table S2).

According to Figure 3A,B, SOD activity in the two sweet potato varieties significantly decreased from 0 to 30 days after heat treatment compared to the activity in CK0. The SOD activity was observed to increase at the beginning of storage and then decrease at the later stage of storage. The heat treatment for P32 and Xinxiang resulted in 8.52% and 29.00% lower SOD activity than in CK0 at 30 days. Moving on to the APX activity (Figure 3C,D), both P32 and Xinxiang with heat treatment showed a significant increase in APX activity at day 0 and day 7 compared to CK0. For P32 in CK0 and HT0, the APX activity peaked at day 0 at 109.17 U·g^−1^ and 125.65 U·g^−1^, respectively. Thus, heat treatment can increase the APX activity of sweet potato tuberous roots. As shown in Figure 3E,F, the PPO activity of P32 and Xinxiang decreased after heat treatment compared to CK0. The PPO activity in all treatments fluctuated between 10.93 U·g^−1^ (Xinxiang HT0, day 15) and 16.85 U·g^−1^ (P32 CK0, day 7). And the differences between the HT0 and CK0 were not significant. Interestingly, the PPO activity of Xinxiang HT0 was higher than that of CK0 at day 0. The minimum PPO activities were reached at 12.62 (P32) and 11.00 (Xinxiang) for HT0 and 11.62 (P32) and 10.93 (Xinxiang) for CK0. The POD activity of P32 progressively increased during the whole storage period (Figure 3G). Both P32 HT0 and P32 CK0 reached the maximum values of 239.42 U·g^−1^ and 261.48 U·g^−1^, respectively, at 60 days. It is worth mentioning that Xinxiang HT0 and CK0′s POD enzyme activities had low values, which fluctuated from 7.16 U·g^−1^ (HT0, day 7) to 13.93 U·g^−1^ (CK0, day 15) (Figure 3H).

These results demonstrate that the activities of the antioxidant enzymes SOD, PPO, and POD were weakened after heat treatment in tuberous roots of sweet potato, except for the enhanced APX activity. These findings differ from those reported by Pan et al. [61], who observed that the heat treatment of sweet potato at 45 °C for 3 h activated the activities of Catalase (CAT) and SOD, effectively blocking the accumulation of superoxide free radicals and hydrogen peroxide. Other studies have reported that appropriate heat treatment of cucumber could increase the APX activity, thereby enhancing the metabolic rate of hydrogen peroxide [62]. Heat treatment inhibited the activities of POD and PPO during the storage of sweet potato tuberous roots, a phenomenon also observed in fruits and vegetables such as persimmon [63] and grape [64]. 

#### 3.2.3. DPPH Radical Scavenging Rate 

**Figure 4:**
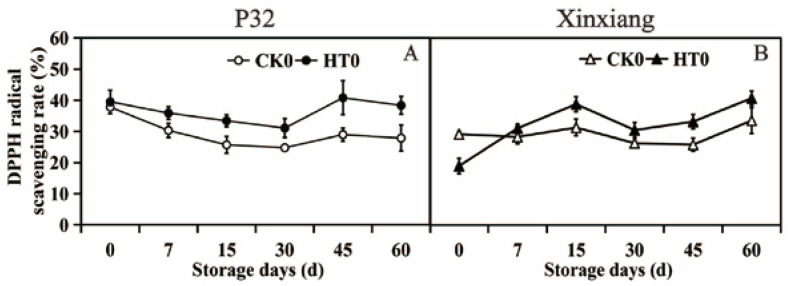
DPPH radical scavenging rate (**A**,**B**) in P32 and Xinxiang tuberous roots stored for 0, 7, 15, 30, 45 and 60 days at 13 °C and 85% RH. CK0 and HT0 mean the sweet potato chips not inoculated with *R. stolonifer*. Significant differences (*p* < 0.05) between means are not indicated by letters above the broken lines, but are presented in the Supplementary Materials with a three-line table.

Heat treatment can increase the DPPH radical scavenging rate of P32 and Xinxiang. The DPPH radical scavenging rate reached the maximum value of 40.81% on the 45th day after heat treatment in P32, surpassing CK0 by 1.41 times. The maximum value of 37.80% in P32 CK0 was at day 0, followed by a downward trend, possibly due to the continuous decline in antioxidant capacity in sweet potatoes with prolonged storage time. On the 60th day, the DPPH radical scavenging rate of Xinxiang reached its maximum values of 40.70% and 33.54%, with HT0 exceeding CK0 by 1.21 times. However, it is puzzling that the DPPH radical scavenging rate of Xinxiang HT0 significantly decreased, being 0.65 times that of CK0 at day 0. In summary, heat treatment did improve the DPPH radical scavenging ability of sweet potato during storage, but for Xinxiang, there was a short-term decline after heat treatment. Despite the decreased activity of antioxidant enzymes such as SOD, POD and PPO after heat treatment, the DPPH radical scavenging rate of sweet potato tuberous roots still increased significantly (Figure 4 and Table S3). This may be attributed to the increased content of ascorbic acid and flavone in APX and non-enzymatic systems [65].

#### 3.2.4. Textural Characteristics 

P32 and Xinxiang exhibited similar texture characteristics, including hardness, springiness, gumminess, chewiness and adhesiveness, under two different treatments, which were increased after heat treatment of P32 and Xinxiang. Compared to CK0, the texture indexes of sweet potato tuberous roots significantly decreased immediately after heat treatment at day 0. However, these indexes were significantly higher after day 7, and the adhesiveness of P32 HT0 and Xinxiang HT0 remained significantly higher throughout the whole storage period. However, heat treatment did not significantly change the cohesiveness of P32 and Xinxiang during the 60-day storage period (Table 1).

The TPA clearly showed that the quality of sweet potato is affected by heat treatment. Therefore, after heat treatment, the texture characteristics of sweet potato during storage can be significantly enhanced to ensure the edible quality. This improvement has the potential to boost sales of sweet potatoes, strengthen consumer preferences, and play a crucial role in generating sales profits [66]. 

### 3.3. Soft Rot Resistance of Swee Potato and Physiological Changes after Heat Treatment

#### 3.3.1. Sweet Potato Soft Rot Resistance 

**Figure 5:**
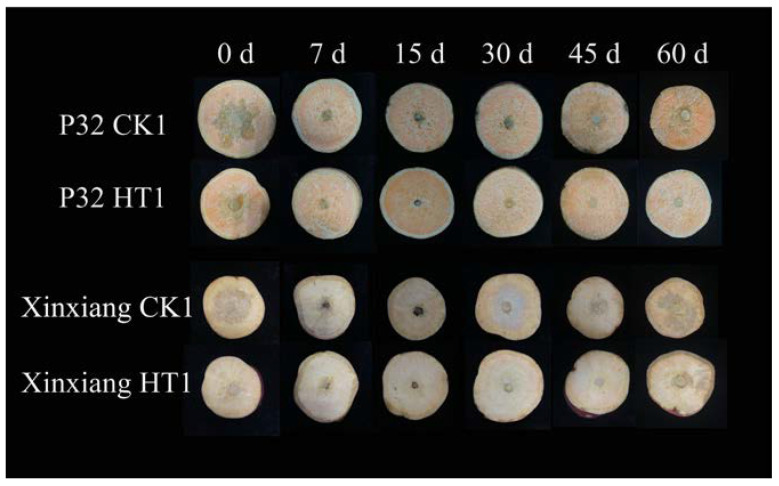
The disease status of sweet potato chips inoculated with *R. stolonifer* for 21 h.

The effects of heat treatment of P32 and Xinxiang in CK1 and HT1 on the resistance of sweet potato chips to *R. stolonifer* during different storage periods were examined. We found that the diameter of disease spots initially decreased and then increased during the 60-day period. We hypothesized that the variation in the disease spot size might be attributed to the delayed activation of some defense mechanisms in sweet potato immediately after heat treatment. There was a time delay, and the effectiveness of these defense mechanisms gradually declined with the extension of the storage period. After heat treatment, the diameters of the disease spots on P32 and Xinxiang were significantly smaller than those on the CK1 samples, indicating that heat treatment effectively inhibits the spread of soft rot (Figure 5).

#### 3.3.2. Antioxidant Enzyme Activity 

**Figure 6:**
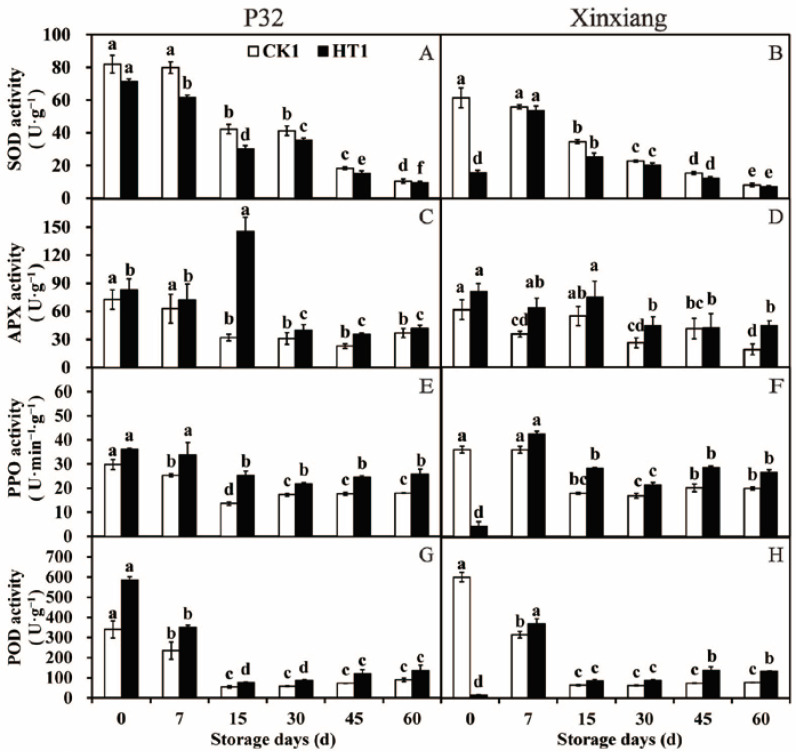
SOD activity (**A**,**B**), APX activity (**C**,**D**), PPO activity (**E**,**F**) and POD activity (**G**,**H**) in P32 and Xinxiang roots stored for 0, 7, 15, 30, 45 and 60 days at 13 °C and 85% RH. CK1 and HT1 mean the sweet potato chips inoculated with *R. stolonifer*. Significant differences (*p* < 0.05) between means are indicated by letters above the histogram bars.

In this section, the changes in antioxidant enzyme activity in sweet potato chips derived from the tuberous roots of P32 and Xinxiang under different storage periods after heat treatment, and subsequently inoculated with *R. stolonifer* for 72 h, were investigated. Over the storage period of P32 and Xinxiang, the SOD activity showed a significant downward trend. The SOD activity of P32 HT1 and Xinxiang HT1 was significantly lower than that of CK1. Interestingly, the SOD activity of Xinxiang HT1 decreased by 0.74 times compared to CK1 (Figure 6A,B and Table S4). There was no general trend in the APX activity of P32 and Xinxiang during the 60-day storage period. Strangely, the APX activity of P32 HT1 sharply increased, reaching a maximum of 145.24 U·g^−1^ at 15 days, significantly higher than that of CK1. Interestingly, the APX activity of Xinxiang HT1 and CK1 fluctuated steadily throughout the whole storage period (Figure 6C,D). During the 60-day storage period, the PPO activity of P32 and Xinxiang generally showed a decreasing tendency. However, the PPO activity of Xinxiang HT1 sharply decreased by 0.88 times compared to CK1 at day 0. The PPO activity of P32 and Xinxiang fluctuated from 13.62 U·g^−1^ (P32 CK1, 15 days) to 28.23 U·g^−1^ (Xinxiang HT1, 15 days) during the period from 15 to 60 days (Figure 6E,F). The trend in POD activity was similar to that of the PPO activity, with heat treatment significantly increasing it compared to that of CK1, but this activity steadily fluctuated from 54.23 U·g^−1^ (P32 CK1, 15 days) to 138.18 U·g^−1^ (Xinxiang HT1, 45 days) during the period from 15 to 60 days. Strangely, the POD activity of Xinxiang HT1 sharply decreased by 0.98 times compared to that of CK1 at day 0, reaching a maximum of 368.23 U·g^−1^ at day 7 (Figure 6G,H). 

In conclusion, heat treatment significantly increased the activities of APX, PPO and POD in P32 and Xinxiang inoculated with *R. stolonifer*. However, these activities in Xinxiang HT1 showed a significant decrease compared to CK1 at day 0. Additionally, heat treatment significantly decreased the SOD activity in both P32 and Xinxiang. In general, heat treatment enhanced the activity of antioxidant enzymes and the disease resistance of sweet potato infected by *R. stolonifer*. Wang et al. [67] reported similar results and showed that treatment with hot air at 43 °C for 3 h effectively inhibited the infection degree of loquat inoculated with *Colletotrichum acutatum*, and the POD and PPO activities of loquat were increased. In this study, the heat treatment increased APX, POD, and PPO activities and enhanced the capacity of sweet potato to scavenge free radicals when infected by *R. stolonifer*. The elevated activities of POD and PPO, which are integral components of the phenylpropane metabolic pathway, suggest heat treatment increased phenylpropane metabolism in sweet potatoes after *R. stolonifer* infection. The phenylpropane metabolism contributes to the production of lignin, thickening cell walls, and improving cellular physical barriers Additionally, it further synthesizes flavonoid phytoprotectin and inhibits the growth of pathogenic bacteria [68,69].

#### 3.3.3. Cell Wall Degrading Enzyme Activity 

**Figure 7:**
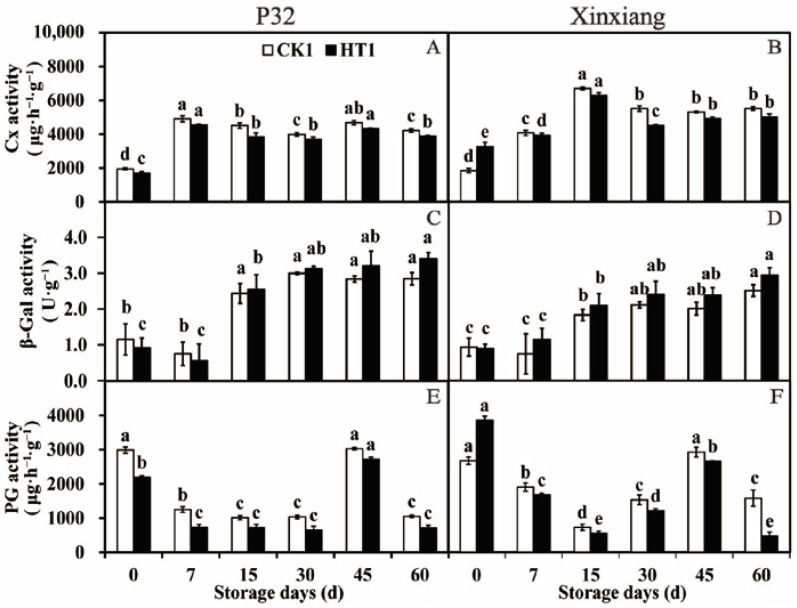
Cx activity (**A**,**B**), β-Gal activity (**C**,**D**) and PG activity (**E**,**F**) in P32 and Xinxiang roots stored for 0, 7, 15, 30, 45 and 60 days at 13 °C and 85% RH. CK1 and HT1 mean the sweet potato chips inoculated with *R. stolonifer*. Significant differences (*p* < 0.05) between means are indicated by letters above the histogram bars.

The changes in cell wall degrading enzyme activity in sweet potato chips, from the sweet potato tuberous roots under different storage periods after heat treatment, inoculated with *R. stolonifer* for 72 h were researched. Cx is a complex enzyme that hydrolyzes cellulose [70]. As shown in Figure 7A and Table S5, both in P32 and Xinxiang, the Cx activity of sweet potatoes was decreased compared to CK1 under different storage times, except at day 0 for Xinxiang. Notably, the Cx activity of Xinxiang HT1 significantly increased by 0.78 times compared to CK1 at day 0, reaching a maximum of 6294.69 U·g^−1^ at 15 days (Figure 7B). β-Gal is a hydrolase that catalyzes the hydrolysis of β-galactoside to monosaccharides [71]. In P32, the β-Gal activity of HT1 was higher than that of CK1, except at day 0 and day 7. In Xinxiang, the β-Gal activity of HT1 was consistently higher than that of CK1, except at day 0. The β-Gal activity of P32 generally decreased first and then increased, while the β-Gal activity of Xinxiang showed an increasing trend during the whole storage period (Figure 7C,D). PG is the main enzyme that degrades pectin in plant cell wall [72]. After heat treatment, the PG activity of P32 and Xinxiang was lower than that of CK1 throughout the 60-day storage period, showing a trend of first decreasing, then increasing and finally decreasing generally. Interestingly, the PG activity of Xinxiang HT1 significantly increased by 0.44 times compared to CK1 at day 0, reaching a minimum 474.19 U·g^−1^ at 60 days (Figure 7E,F).

Thus, heat treatment significantly decreased the activities of Cx and PG in sweet potatoes inoculated with *R. stolonifer* while increasing the β-Gal activity. Overall, the activities of cell wall degrading enzymes were decreased, and the disease resistance of infected sweet potatoes was enhanced after heat treatment. The findings are similar to those of Chopsri et al. [73], which discovered that hot air treatment (at 50 °C for 10 min) effectively inhibited the activities of the banana cell wall degrading enzymes Pectin methylesterase (PME), PG and pectate lyase (PL). In addition, the enzyme activities and gene expression of PME, PG, Cx and β-Gal in cherry tomatoes were effectively inhibited by hot air treatment at 38 °C for 12 h [74]. The PME and PG activities of melons were increased by heat treatment at 45 °C for 3 h and at 50 °C for 2 h [75].

## 4. Conclusions

This study provides evidence that the application of heat treatment (at 35 °C for 24 h) effectively inhibits the growth and reproduction of *R. stolonifer* in vitro. Additionally, the healthy sweet potato treated at 35 °C for 24 h did not lead to a significant increase in antioxidant enzymes, but the DPPH radical scavenging rate increased significantly, which better maintained the nutritional quality and texture characteristics of sweet potato during storage. Moreover, after heat treatment, the resistance of sweet potato to soft rot increased during the 60-day storage period. Overall, these results indicate that heat treatment may be a simple and attractive physical method to delay infection by sweet potato soft rot and extend the shelf life of sweet potato. 

## Figures and Tables

**Table 1:** Texture characteristics of P32 and Xinxiang (at 13 °C and 85% RH).

Texture	Treatment	Storage Days					
		0	7	15	30	45	60
**Hardness**	**P32 CK0**	97.59 ± 0.86 b	91.93 ± 4.04 c	93.72 ± 1.03 bc	102.57 ± 1.43 a	104.8 ± 2.59 a	104.55 ± 0.89 a
	**P32 HT0**	92.05 ± 1.34 c	95.78 ± 4.17 c	97.71 ± 3.69 c	106.96 ± 0.78 b	116.65 ± 1.7 a	108.84 ± 0.87 b
	**Xinxiang CK0**	115.72 ± 4.05 a	101.2 ± 8.55 b	99.23 ± 5 b	107.3 ± 5.15 ab	110.12 ± 0.95 ab	104.2 ± 2.77 ab
	**Xinxiang HT0**	110.8 ± 4.05 a	105.98 ± 6.25 a	105.76 ± 1.17 a	113.71 ± 3.06 a	113.82 ± 1.16 a	111.27 ± 1.44 a
**Adhesiveness**	**P32 CK0**	20.28 ± 2.04 ab	21.72 ± 0.99 ab	11.25 ± 1.18 c	12.58 ± 2.61 c	18.57 ± 1.66 b	22.94 ± 1.23 a
	**P32 HT0**	20.91 ± 1.93 bc	25 ± 1.13 ab	13.04 ± 2.26 d	17.43 ± 1.74 c	21.45 ± 1.7 bc	27.07 ± 2.32 a
	**Xinxiang CK0**	11.91 ± 1.33 bc	10.31 ± 0.48 bc	9.68 ± 0.34 bc	8.16 ± 0.42 c	13.42 ± 2.6 ab	16.04 ± 2.72 a
	**Xinxiang HT0**	14.87 ± 0.48 ab	13.58 ± 2 bc	10.57 ± 2.17 c	10.81 ± 1.29 c	16.47 ± 0.89 ab	17 ± 1.15 a
**Cohesiveness**	**P32 CK0**	0.22 ± 0 a	0.23 ± 0.02 a	0.24 ± 0.01 a	0.21 ± 0.01 a	0.22 ± 0 a	0.23 ± 0.01 a
	**P32 HT0**	0.24 ± 0 a	0.25 ± 0.02 a	0.24 ± 0.01 a	0.24 ± 0.01 a	0.22 ± 0.01 a	0.23 ± 0.01 a
	**Xinxiang CK0**	0.27 ± 0.03 a	0.2 ± 0.03 b	0.21 ± 0.02 b	0.2 ± 0.02 b	0.21 ± 0.01 b	0.22 ± 0.02 b
	**Xinxiang HT0**	0.22 ± 0.02 a	0.22 ± 0.01 a	0.22 ± 0.01 a	0.21 ± 0.02 a	0.22 ± 0.01 a	0.22 ± 0 a
**Springiness**	**P32 CK0**	6.56 ± 0.12 a	6.31 ± 0.17 ab	5.58 ± 0.18 bc	5.19 ± 0.4 c	5.99 ± 0.14 ab	5.95 ± 0.65 abc
	**P32 HT0**	6.33 ± 0.35 ab	6.58 ± 0.13 ab	6.18 ± 0.21 ab	5.78 ± 0.08 b	6.91 ± 0.06 a	6.96 ± 0.74 a
	**Xinxiang CK0**	7.62 ± 0.35 a	5.76 ± 0.28 b	5.62 ± 0.33 bc	5.08 ± 0.29 c	5.91 ± 0.11 b	5.87 ± 0.09 b
	**Xinxiang HT0**	6.37 ± 0.23 a	6.07 ± 0.24 ab	6.49 ± 0.18 a	5.58 ± 0.28 b	6.39 ± 0.29 a	6.03 ± 0.14 ab
**Gumminess**	**P32 CK0**	22.02 ± 0.21 ab	21.96 ± 2.88 ab	20.59 ± 0.28 b	20.24 ± 0.65 b	24.01 ± 0.46 a	21.45 ± 0.45 ab
	**P32 HT0**	21.53 ± 0.85 a	23.57 ± 2.73 a	22.06 ± 0.37 a	23.57 ± 0.68 a	24.71 ± 1.58 a	23.81 ± 0.33 a
	**Xinxiang CK0**	25.78 ± 3.08 a	21.06 ± 0.4 b	20.94 ± 0.8 b	21.38 ± 1.56 b	22.66 ± 0.55 ab	22.92 ± 0.16 ab
	**Xinxiang HT0**	23.99 ± 1.36 a	23.05 ± 0.93 a	23.67 ± 0.91 a	24 ± 0.53 a	25.15 ± 0.78 a	24.54 ± 0.8 a
**Chewiness**	**P32 CK0**	144.61 ± 2.07 a	139.61 ± 8.35 ab	127.69 ± 6.81 bc	121.32 ± 8.59 c	144.03 ± 1.27 a	150.79 ± 4.25 a
	**P32 HT0**	136.67 ± 9.7 b	153.98 ± 22 ab	135.53 ± 1.91 b	143.52 ± 5.54 ab	170.89 ± 12.44 a	166.89 ± 11.11 a
	**Xinxiang CK0**	202.39 ± 24 a	140.09 ± 13.1 b	130.72 ± 12.3 b	114.25 ± 19.01 b	139.54 ± 2.53 b	144.29 ± 2.04 b
	**Xinxiang HT0**	153.42 ± 13.06 ab	153.71 ± 4.36 ab	147.24 ± 11.54 ab	133.73 ± 11.2 b	161.18 ± 8.47 a	155.08 ± 3.4 ab

Data are expressed as means of three replicates ± SD. Values in a column followed by different letters are significantly different (*p* < 0.05).

## Data Availability

All of the supporting data are included within the article and its supplementary files.

## Supplementary Materials

The following supporting information can be downloaded at: https://www.mdpi.com/article/10.3390/foods12234352/s1, Figure S1: The calculation of TPA test parameters; Table S1: Flavone, starch, soluble sugar and protein contents; Table S2: SOD, APX, PPO and POD activities; Table S3: DPPH radical scavenging rate; Table S4: SOD, APX, PPO and POD activities of sweet potato infected by *R. stolonifer*; Table S5: Cx, β-Gal and PG activities of sweet potato infected by *R. stolonifer*.
