# Supplementary material for: Effect of Heat Treatment on the Quality and Soft Rot Resistance of Sweet Potato during Long-Term Storage
Source: Foods. 2023 Dec 2;12(23):4352. doi: 10.3390/foods12234352 (PMC10706704; doi:10.3390/foods12234352)
Supplement: Supplementary file 1 [file foods-12-04352-s001.zip › foods-2723427-supplementary.pdf]

Effect of heat treatment on sweetpotato quality and soft rot resistance during long-term storage

Jifeng Wu[1,†], Jingzhen Zhang[1,†], Wenrong Ni[1], Ximing Xu[1], Melvin Sidikie George[2] and Guoquan Lu[1,*]

[1]Institute of Root and Tuber Crops, The Key Laboratory for Quality Improvement of Agricultural Products of Zhejiang Province, College of Advanced Agricultural Sciences, Zhejiang A&F University, Hangzhou 311300, China

[2]Crop Science Department, Njala University, Njala Campus. Private Mail Bag, Freetown, 999127, Sierra Leone

[*]Correspondence: lugq@zafu.edu.cn

[†]These authors contributed equally to this work.

Supporting Information

Figure S1. The calculation of TPA test parameters. 3
Table S1. Flavone, starch, soluble sugar and protein contents. 4
Table S2. SOD, APX, PPO and POD activities. 5
Table S3. DPPH radical scavenging rate. 6
Table S4. SOD, APX, PPO and POD activities of sweet potato infected by *R. stolonifer*. 7
Table S5. Cx, β-Gal and PG activities of sweet potato infected by *R. stolonifer*. 8

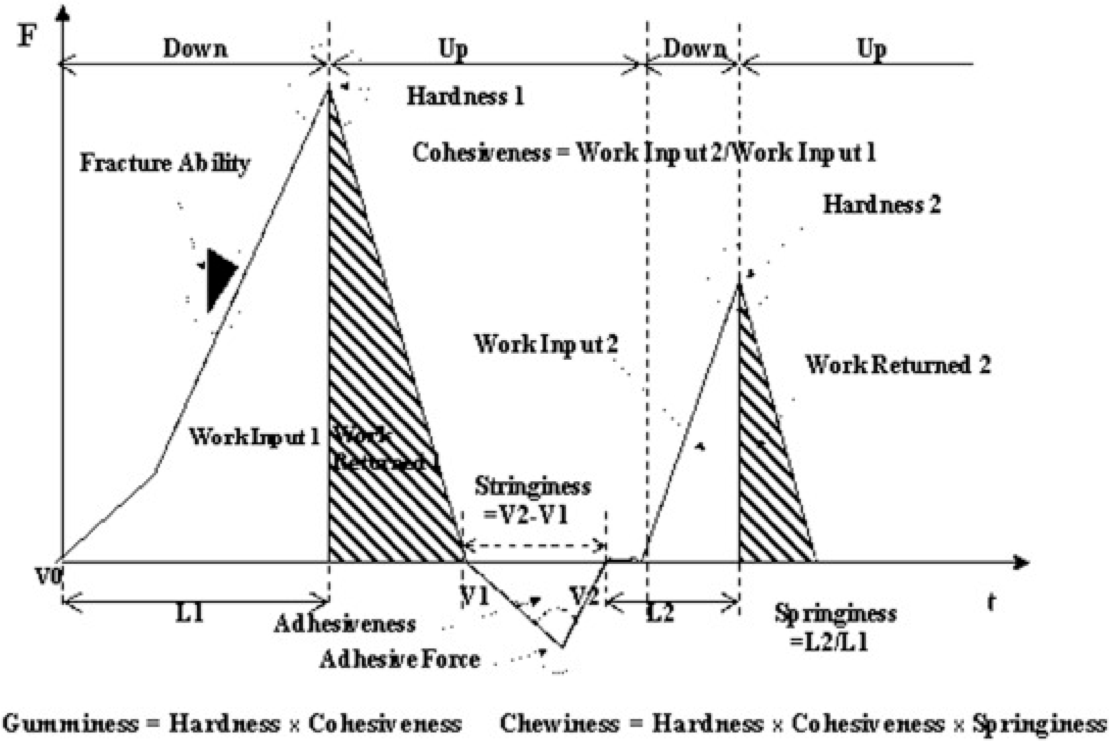

Figure S1. The calculation of TPA test parameters.

Table S1. Flavone, starch, soluble sugar and protein contents.

Type	Treatment	Storage days(d)					
		0d	7d	15d	30d	45d	60d
Flavone	P32 CK0	0.5±0.01a	0.39±0b	0.37±0c	0.49±0a	0.39±0b	0.5±0.02a
	P32 HT0	0.5±0a	0.4±0d	0.41±0c	0.47±0b	0.39±0d	0.51±0.01a
	Xinxiang CK0	0.67±0d	0.56±0f	0.65±0.01e	0.76±0b	0.69±0c	0.81±0a
	Xinxiang HT0	0.61±0d	0.58±0e	0.65±0c	0.73±0b	0.84±0a	0.73±0b
Starch	P32 CK0	61.3±0.05b	60.7±0.47bc	60.39±0.22bc	63.06±0.35a	60.2±0.19c	62.44±0.81a
	P32 HT0	61.2±0.45bc	60.53±0.22cd	60.07±0.42d	64.91±0.24a	61.93±0.22b	65.09±0.92a
	Xinxiang CK0	72.07±0.13c	71±0.44d	71.45±0.2cd	76.06±0.42a	75.21±0.23b	75.86±0.41ab
	Xinxiang HT0	71.17±0.42d	71.16±0.25d	72.01±0.23c	77.44±0.3b	77.62±0.14b	78.75±0.17a
Soluble sugar	P32 CK0	12.81±0.16d	14.33±0.24b	14.75±0.13b	13.76±0.1c	15.7±0.14a	14.59±0.28b
	P32 HT0	13.98±0.12d	15.07±0.13b	15.3±0.07b	14.5±0.13c	15.94±0.15a	13.43±0.32e
	Xinxiang CK0	12.01±0.11c	13.14±0.11c	12.36±0.07a	10.53±0.11b	11.82±0.23d	10.01±0.18a
	Xinxiang HT0	12.1±0.12e	13.1±0.07a	12.5±0.06c	10.33±0.19c	10.69±0.08d	11.78±0.03b
Protein	P32 CK0	3.26±0.02a	3.06±0.06b	3.41±0.04a	2.95±0.02bc	2.84±0.04cd	2.76±0.18d
	P32 HT0	2.73±0.01d	2.56±0.02e	3.07±0.01c	3.17±0.03b	3.31±0.01a	3.12±0.09bc
	Xinxiang CK0	1.26±0.02c	1.28±0.01c	1.45±0.03a	1.4±0.02b	1.16±0.02d	1.48±0.02a
	Xinxiang HT0	1.2±0.04e	1.79±0.05a	1.48±0.01c	1.49±0.04c	1.33±0.03d	1.71±0.04b

Table S2. SOD, APX, PPO and POD activities.

Type	Treatment	Storage days(d)					
		0d	7d	15d	30d	45d	60d
SOD	P32 CK0	32.35±1.07c	37.4±1.67b	35.83±1.52b	44.81±1.96a	16.38±0.95d	10.74±1.35e
	P32 HT0	22.81±2.22c	30.88±1.79b	30.41±2.24b	40.99±1.67a	15.35±1.65d	9.22±1.22e
	Xinxiang CK0	21.06±1.27c	28.64±1.7b	31.46±3.59ab	34.83±0.72a	11.74±0.64d	8.25±1.01d
	Xinxiang HT0	14.98±0.37b	24.43±0.57a	22.47±0.54a	24.73±2.41a	11.5±2.37b	4.27±1.81c
APX	P32 CK0	109.17±7.3a	73.39±8.31b	23.39±6.87d	40.71±11.84cd	55.24±1.22c	32.68±7.16d
	P32 HT0	125.65±22.64a	101.13±47.24ab	28.75±1.77c	42.2±13.23c	58.04±5.18bc	29.46±4.87c
	Xinxiang CK0	47.68±5.13c	92.2±7.36a	63.04±8.14b	40.36±4.32cd	38.57±2.28cd	28.15±3.83d
	Xinxiang HT0	60.89±15.9bc	112.68±22.91a	74.23±16.99b	43.15±6.95bc	43.39±9.09bc	32.2±8.77c
PPO	P32 CK0	15.85±0.29a	16.85±1a	12.62±1.28c	14.9±1.1ab	13.77±0.78bc	16.48±0.57a
	P32 HT0	16.13±0.12a	15.95±2.19a	11±1.12b	14.1±2.1ab	12.28±1.4ab	14.97±2.07a
	Xinxiang CK0	13.07±2.06ab	13.83±0.83ab	11.62±1.02b	13.72±0.85ab	13.47±0.59ab	14.28±0.52a
	Xinxiang HT0	16.08±2.46a	13.07±1.68ab	10.93±0.7b	12.07±2.95ab	13.08±1.11ab	12.95±2.11ab
POD	P32 CK0	21.22±0.63e	28.21±1.6e	73.65±11.91d	112.16±9.74c	197.37±4.04b	261.48±15.75a
	P32 HT0	22.67±1.65d	28.12±1.58d	55.39±26.86cd	91.52±22.3c	171.43±35.82b	239.42±45.09a
	Xinxiang CK0	9.02±1.46c	12.29±1.99ab	13.93±1.52a	10.6±0.7bc	10.49±0.29bc	11.32±0.79abc
	Xinxiang HT0	10.41±2.4a	11.22±3.07a	13.2±2.22a	9.49±1.79a	9.36±1.41a	11.15±0.97a

Table S3. DPPH radical scavenging rate.

Type	Treatment	Storage days(d)					
		0d	7d	15d	30d	45d	60d
DPPH radical scavenging rate	P32 CK0	37.8±0.76ab	30.34±2.25ab	25.71±2.71b	28.63±2.78b	43.41±3.82a	36.32±13.55ab
	P32 HT0	39.52±3.74a	35.9±2.06ab	33.43±1.89ab	31.09±3.03b	40.81±5.43a	38.36±2.88ab
	Xinxiang CK0	29.2±1.55bc	28.3±0.09cd	31.33±0.91ab	26.3±1.63d	25.81±0.43d	33.54±1.05a
	Xinxiang HT0	18.97±2.48c	31.08±1.27b	38.8±2.4a	30.5±2.44b	33.21±2.26b	40.7±2.26a

Table S4. SOD, APX, PPO and POD activities of sweet potato infected by *R. stolonifer*.

Type	Treatment	Storage days(d)					
		0d	7d	15d	30d	45d	60d
SOD	P32 CK1	82.03±5.41a	79.89±3.5a	42.32±2.91b	41.25±2.97b	18.35±0.8c	10.51±1.29d
	P32 HT1	71.43±1.54a	61.61±1.44b	30.03±2.24d	35.4±1.52c	15.25±1.58e	9.51±0.93f
	Xinxiang CK1	61.44±6.01a	55.9±1.19a	34.7±1.18b	22.93±0.64c	15.61±0.89d	8.31±1.11e
	Xinxiang HT1	15.82±1.47d	53.54±2.92a	25.25±2.56b	20.26±1.47c	12.4±1.01d	7.31±0.62e
APX	P32 CK1	72.68±10.39a	62.98±15.35a	32.14±3.39b	31.07±6.17b	23.04±2.41b	36.96±4.83b
	P32 HT1	83.21±11.48b	72.02±17.26b	145.24±15.24a	39.64±6.35c	35.36±1.63c	41.96±3.22c
	Xinxiang CK1	61.48±10.58a	35.54±3.04cd	54.82±10.32ab	26.49±5.3cd	41.43±10.94bc	19.29±5.88d
	Xinxiang HT1	81.01±8.55a	63.89±9.97ab	75.18±16.7a	44.52±9.3b	42.68±14.58b	44.64±5.39b
PPO	P32 CK1	29.82±2.11a	25.33±0.66b	13.62±0.7d	17.3±0.57c	17.65±0.64c	17.95±0.14c
	P32 HT1	36.15±0.39a	33.73±5.21a	25.28±1.73b	21.77±0.46b	24.47±0.74b	25.72±2.23b
	Xinxiang CK1	35.93±1.36a	35.87±1.41a	17.9±0.44bc	16.85±1.03c	20.17±1.59b	19.87±0.61b
	Xinxiang HT1	4.28±1.88d	42.42±1.19a	28.23±0.42b	21.35±1.01c	28.6±0.6b	26.57±1.07b
POD	P32 CK1	340.81±42.89a	234.81±42.49b	54.23±5.86c	58.91±2.06c	72.74±1.28c	89.74±9.76c
	P32 HT1	586.84±13.75a	351.33±10.58b	75.92±3.71d	85.47±6.37d	120.02±20.07c	136.45±26.65c
	Xinxiang CK1	582.65±23.16a	315.26±17.03b	64.83±2.86c	63.63±1.22c	74.55±1.89c	78.01±0.63c
	Xinxiang HT1	14.91±1.54d	368.23±25.9a	84.25±9.1c	87.11±5.75c	138.18±16.84b	132.05±4.6b

Table S5. Cx, β-Gal and PG activities of sweet potato infected by *R. stolonifer*.

Type	Treatment	T					
		0d	7d	15d	30d	45d	60d
Cx	P32 CK1	1949.64±56.61d	4908.29±182.62a	4506.5±160.51b	3987.22±106.14c	4680.86±125.31ab	4217.31±102.8c
	P32 HT1	1705.62±86.62c	4544.26±59.28a	3825.22±249.92b	3702.02±125.72b	4339.63±29.24a	3884.88±53.46b
	Xinxiang CK1	1837.3±131.54d	4072.38±152.07c	6703.51±86a	5511.92±157.15b	5302.4±54.02b	5512.56±105.74b
	Xinxiang HT1	3272.1±240.63e	3941.24±102.47d	6294.69±154.97a	4515.23±53.54c	4927.59±99.64b	5036.58±141.44b
β-Gal	P32 CK1	1.15±0.43b	0.75±0.33b	2.43±0.28a	2.99±0.04a	2.84±0.09a	2.84±0.17a
	P32 HT1	0.91±0.28c	0.55±0.47c	2.55±0.41b	3.12±0.07ab	3.2±0.41ab	3.4±0.18a
	Xinxiang CK1	0.93±0.25c	0.75±0.56c	1.83±0.16b	2.11±0.09ab	2.01±0.18ab	2.52±0.16a
	Xinxiang HT1	0.89±0.13c	1.15±0.31c	2.1±0.33b	2.41±0.37ab	2.38±0.22ab	2.95±0.21a
PG	P32 CK1	2986.22±92.57a	1249.22±80.54b	1010.46±63c	1036.71±48.07c	3027.33±34.46a	1051.87±35.84c
	P32 HT1	2191.16±44.17b	732.31±81.43c	726.16±91.21c	651.09±109.51c	2721.88±55.02a	715.65±71.19c
	Xinxiang CK1	2667.39±108.12a	1899.73±122.04b	729.1±90.82d	1526.21±138.1c	2917.92±142.17a	1576.84±236.59c
	Xinxiang HT1	3843.61±121.18a	1668.4±55.18c	547.19±72.66e	1207.13±60.75d	2650.93±13.6b	474.19±113.12e
